# RDM1 promotes neuroblastoma growth through the RAS–Raf–MEK–ERK pathway

**DOI:** 10.1002/2211-5463.12586

**Published:** 2019-01-28

**Authors:** Guojin Xie, Haiyan Wu, Weiluo Cai, Mo Chen, Wending Huang, Wangjun Yan, Yong Chang

**Affiliations:** ^1^ Children^’^s Hospital of Nanjing Medical University Jiangsu China; ^2^ Department of Musculoskeletal Tumor Shanghai Cancer Center Fudan University Shanghai China

**Keywords:** apoptosis, neuroblastoma, RAS–Raf–MEK–ERK pathway, RDM1

## Abstract

Neuroblastoma (NB) is an aggressive cancer that originates in the sympathetic nervous system and primarily affects children. Here, we show that high levels of RAD52 motif containing 1 (RDM1; a protein with similarities to RAD52, which is important for double‐strand DNA repair) are associated with poor clinical outcomes for NB. In addition, RDM1^−/−^ cells exhibited increased sensitivity to cisplatin, a common chemotherapy drug, and disruption of *RDM1* suppressed NB cell proliferation. We also report that loss of RDM1 augmented cell apoptosis and induced cell cycle arrest, and that stable knockdown of *RDM1* significantly inhibited NB tumor growth in a xenograft mouse model. Importantly, we identified that RDM1 promoted cell proliferation via the RAS–Raf–mitogen‐activated protein kinase kinase (MEK)–extracellular signal‐regulated kinase (ERK) signaling pathway. In conclusion, the current study demonstrates a correlation between DNA damage regulator RDM1 and the oncogenic RAS–Raf–MEK–ERK pathway in NB.

**Abbreviations**
DDR: DNA damage response
DSB: double‐strand break
ERK: extracellular signal‐regulated kinase
IHC: immunohistochemistry
MEK: mitogen‐activated protein kinase kinase
NB: neuroblastoma
RDM1: RAD52 motif containing 1
PI: propidium iodide
siRNA: small interfering RNA

Neuroblastoma (NB), a common childhood tumor that originates from the sympathetic nervous system, shows aggressive tumor formation 1, 2. The cancer often begins in early childhood, developing in the neck, chest, abdomen, or spine. NB is responsible for 15% of childhood mortality, and the survival rate for metastatic tumors is only 40% after 5 years 3, 4. Due to the lack of specific biomarkers for NB, there are no efficient treatment approaches.

Of those high risk patients, some sporadic genomic changes have been detected in NB, including *MYCN* 5, *PHOX2B* 6 and *ALK* 7. It was reported that MYCN overexpression occurs in 25–30% of NB cases, and it may work as a predictive biomarker of NB 8. Amplified MYCN forms hetero‐dimers with MAX to act as a transcription factor and induces NB tumor growth 9. ALK is a member of the insulin receptor superfamily of transmembrane receptor tyrosine kinases. Changes in the *ALK* gene are responsible for NBs 7. Although many other genetic abnormalities were demonstrated, they are not considered as good therapy targets because different mutations occur in these genes, and it is therefore difficult to target them for successful therapy. Thus, a better understanding of NB development will help to improve treatment.

The gene encoding RAD52 motif‐containing protein 1 (RDM1) is located at 17q11.2 and belongs to the gene‐binding motif containing family 10. RDM1 is a key regulator involved in the DNA damage repair pathway and RDM1^−/−^ cells increase sensitivity to cisplatin, a common chemotherapy drug 11, 12, 13, 14. One of the most prominent hallmarks of cancer is genomic instability. The repair of double‐strand breaks (DSBs) is mediated by RAD52‐dependent recombination and the genomic integrity resulted from dysfunctional DNA damage response (DDR) signaling in the DNA repair pathways 15. RDM1 was found to have function in lung cancer 16 and papillary thyroid carcinoma 17, but its function in NB progression remains unclear.

Given the potential role of RDM1 in the DNA repair pathways, we found that RDM1 is up‐regulated in NB patient samples and the up‐regulation of RDM1 is correlated with poor clinical prognosis. Moreover, we investigated the effect of RDM1 on NB cell growth, cell apoptosis and the cell cycle. We further evaluated the *in vivo* growth of *RDM1*‐knockdown cells in a mouse xenograft model. Interestingly, knockdown of *RDM1* inactivated the RAS–Raf–mitogen‐activated protein kinase kinase (MEK)–extracellular signal‐regulated kinase (ERK) signaling pathway. Taken together, our findings present a novel insight into the oncogenic role of RDM1 in the development of NB.

## Materials and methods

### Cells and reagents

Neuroblastoma cell lines SH‐SY5Y and SK‐N‐AS were bought from American Type Culture Collection (Manassas, VA, USA). Cell cultures were maintained at 37 °C in a humidified atmosphere consisting of 5% CO_2_.

Antibodies were purchased from: Cell Signaling Technology Inc. (Shanghai, China) [phosphorylated (P) ‐ERK, RAS, P‐BRAF, P‐MEK and poly (ADP‐ribose) polymerase (46D11)]; Proteintech Inc., Shanghai, China (RDM1); Sigma (β‐actin).

### RNA interference of RDM1 and RNA analyses

*RDM1* small interfering RNAs (siRNAs) were selected based on 18. The siRNA sequence is 5′‐UCAGAAGGCUUUGUCAGAUTT‐3′. The siRNA of RDM1 was synthesized by GenePharma Co Inc. (Shanghai, China). Cells were homogenized in 1 mL RNAiso™ Plus lysis buffer (Takara Inc., Shanghai, China). Total RNA was extracted and 2 μg RNA was reverse transcribed into cDNA following the manufacturer's instruction.

### Soft‐agar colony formation assay

Both targeted‐knockdown (si*RDM1*) and control cells were seeded in 96‐well dishes with a density of 5 × 10^3^ cells per well. The amount of dye taken up by the monolayer was quantified in a spectrophotometer or plate reader at 570 nm. The spectrophotometer used in the colony formation assay was the Spectra Max 190 (Molecular Devices Company Inc., Shanghai, China).

### Analysis of apoptosis

Cells were seeded in six‐well plates and treated with the indicated treatments for 72 h. Cells were harvested and washed with cold PBS, followed by incubation with propidium iodide (PI, 100 μg·mL^−1^) and annexin V–Alexa Fluor488 conjugate at room temperature for 15 min. The parameters of 494/518 nm set for annexin V and 535/617 nm for PI were used to analyze the cell apoptosis. PI and annexin V–Alexa Fluor488 were from BD Biosciences Inc. (Shanghai, China).

### Cell cycle analysis

Cells were washed twice with cold PBS, resuspended in cold 70% ethanol and stored at 4 °C overnight. For flow cytometry, cells were washed three times in 3 mL of cold PBS and then stained with a PI solution (50 μg·mL^−1^ PI/50 units·mL^−1^ RNase free) for 20 min at room temperature. Cell cycle analysis was performed using a cell analyzer (BD CantoII Inc., Shanghai, China).

### Western blot

For western blot, an equivalent amount of protein from NB cells was loaded and the immune‐blots were analyzed using primary antibodies specific for RDM1 and P‐ERK overnight at 4 °C. After incubation with fluorescently labeled secondary antibody, it was visualized with a LI‐COR Odyssey Infrared Imaging System (Lincoln, NE, USA).

### Immunohistochemistry

Dissected tumors from patients and mice were fixed with 4% paraformaldehyde for 3 days and were then dehydrated through a graded series of ethanol, embedded in paraffin and sectioned at 4 μm.

After a graded series of ethanol, the slides were incubated with RDM1 and P‐ERK antibodies overnight at 4 °C. Sections were finally stained with 3,3‐diaminobenzidine tetrahydrochloride after incubation with the secondary antibody. We observed the immunohistochemistry (IHC) images using a Leica microscope (Leica Inc., Shanghai, China, DM4000b).

### Mouse xenograft tumor model

Five female BALB/c nude mice at the age of 5 weeks were prepared. Sh*RDM1* and control cells were implanted into the mice subcutaneously on both flanks at 2 × 10^6^ cells. Four weeks after injection, mice bearing tumors were euthanized for the assessment of tumor size and immunohistological examination. All animal studies were performed in accordance with the National Institutes of Health's *Guide for the Care and Use of Laboratory Animals*, with the approval of the Animal Research Committee of the Affiliated Children's Hospital of Nanjing Medical University, Jiangsu Province, China.

### Clinical tumor samples

Neuroblastoma samples were collected from The Affiliated Children's Hospital of Nanjing Medical University. The patients were selected according to the following criteria: all patients were diagnosed and confirmed by pathology; patients with NB had no other cancers and no preoperative chemotherapy or radiotherapy was administered to the cancer patients.

All samples were collected in accordance with ethical guidelines, and written informed consent was received. All patients were approached based on approved ethical guidelines. The study methodologies conformed to the standards set by the Declaration of Helsinki. The research protocol and consent program were approved by the Affiliated Children's Hospital of Nanjing Medical University Medical Institutional Ethical Committee.

### Analysis of human lung cancer tumor samples

All NB samples (*n* = 25) were from surgical resection. All clinical samples were devoid of personal information. The staining intensity was evaluated on a scale of 0–3, and was rated as negative (−), weak staining (+), moderate/strong staining (+ +) and very strong staining (+ + +).

### Statistical analysis

Student's *t* test was performed to obtain the statistical significance. A *P* value < 0.05 was considered as a significant difference.

## Results

### RDM1 is up‐regulated in human NB samples

**Figure 1:**
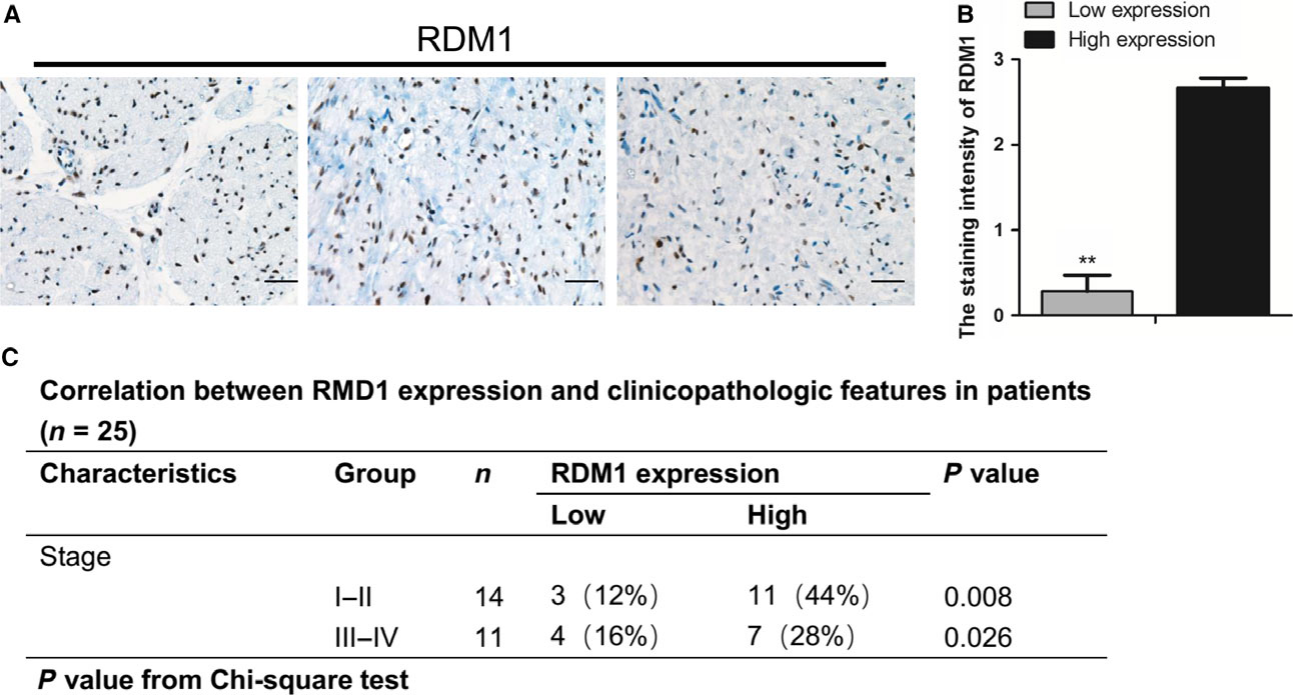
RDM1 is up‐regulated in human NB samples. (A) IHC analysis of RDM1 in clinical NB samples. The results indicated that RDM1 was significantly overexpressed in NB tissues. Scale bar: 25 μm (magnification: ×40). (B) Statistical analysis of the staining intensity of RDM1 in (A) (low expression, *n* = 7; high expression, *n* = 11; **P < 0.001). Data represent means ± SEM, as determined by Student's *t* test. (C) The correlation between RDM1 expression and clinicopathological features of different patients (*n* = 25, *P* < 0.05).

The expression of RDM1 was examined in NB samples from patients, and IHC results indicated that RDM1 was significantly overexpressed in NB tissues (Fig. 1A,B). In addition, we explored whether the expression of RDM1 was associated with NB patients’ prognosis. Statistical analyses indicated that up‐regulation of RDM1 was significantly correlated with tumor stage (Fig. 1C).

### Knocking down *RDM1* inhibits cellular proliferation

**Figure 2:**
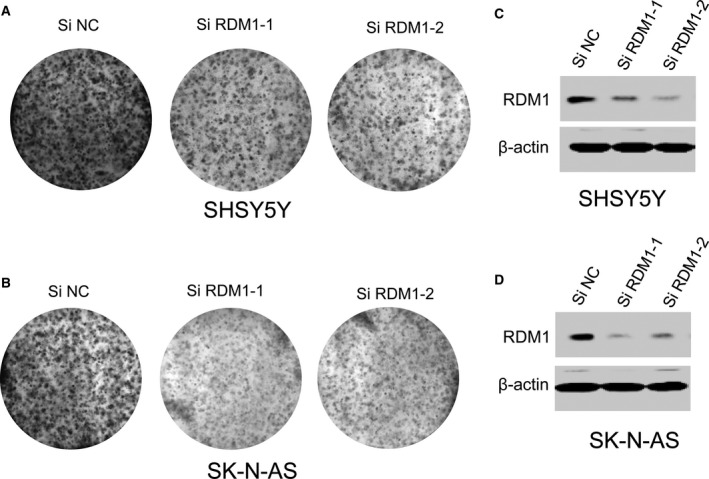
RDM1 promotes NB cell growth. (A,B) Soft‐agar colony formation for SHSY5Y (A) and SK‐N‐AS (B) cells at day 5 after transfection with scrambled siRNA (negative control, siN) and RDM1‐siRNA‐1, siRNA‐2. The data showed knockdown of *RDM1* resulted in a significant decrease in colony number. (C,D) Western blot showed that RDM1 siRNA‐transfected SHSY5Y (C) and SK‐N‐AS (D) cells exhibited decreased RDM1. Actin served as the loading control in the western blot experiments.

RDM1 is reported to be an essential factor that regulates cell proliferation. Next, we wanted to determine whether RDM1 affects NB cell proliferation because RDM1 is highly expressed in NB samples by bioinformatics analysis and IHC study. We firstly knocked down *RDM1* by siRNA in both SH‐SY5Y and SK‐N‐AS cell lines and then stained the cells with crystal violet to count colony number. As shown in Fig. 2A,B, knockdown of *RDM1* resulted in a significant decrease in colony number. The *RDM1* knockdown efficiency was confirmed in both SH‐SY5Y (Fig. 2C) and SK‐N‐AS (Fig. 2D) cell lines. Taken together, these data suggest that RDM1 is a positive regulator of NB cells.

### Lack of RDM1 augments cell apoptosis and induces cell cycle arrest

**Figure 3:**
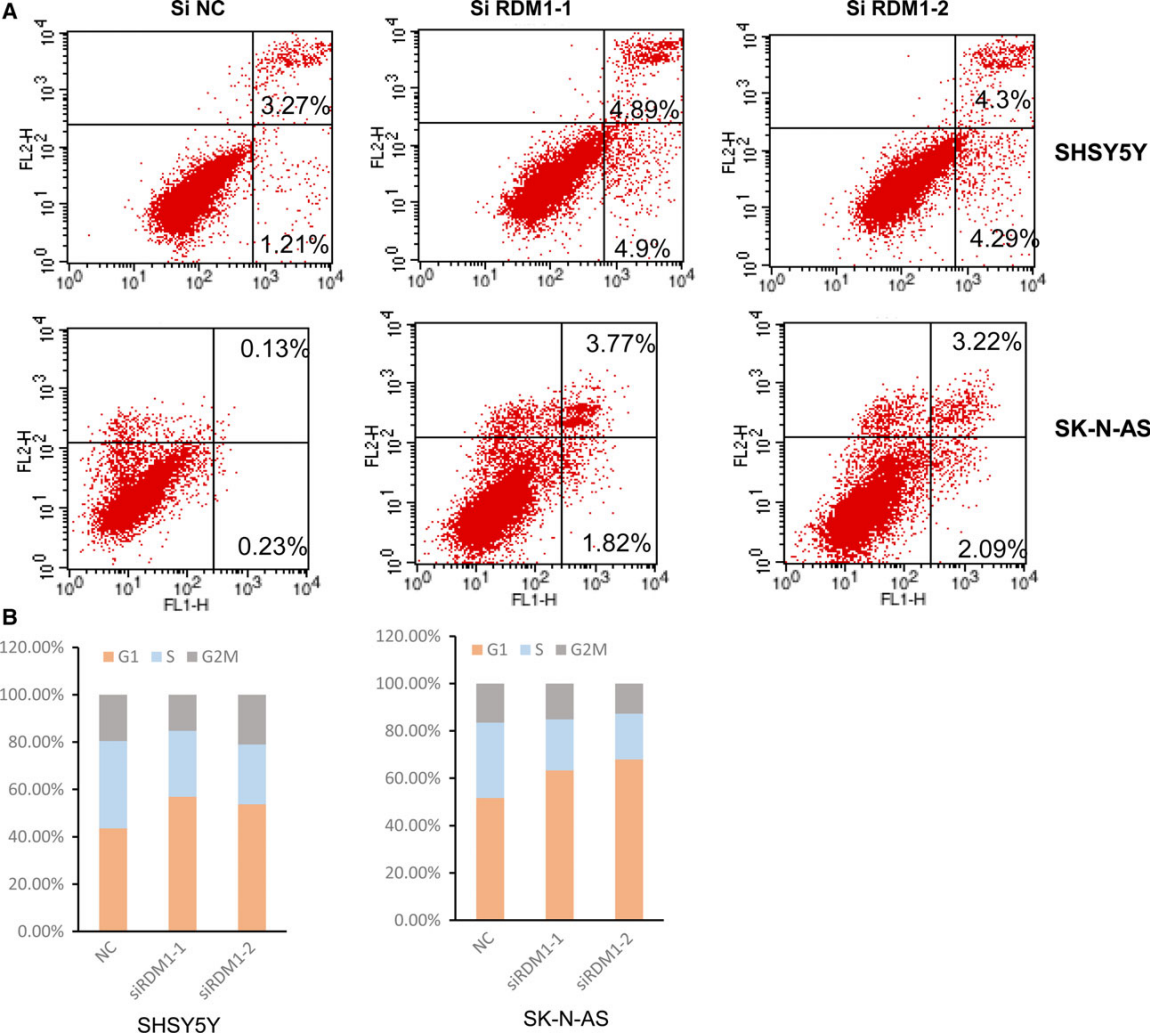
Lack of RDM1 augments the cell apoptosis and induces cell cycle arrest. (A) Flow cytometry analysis of apoptosis in SHSY5Y (A) and SK‐N‐AS (B) cells after transfection with scrambled siRNA (negative control, siN) and RDM1‐siRNA‐1, siRNA‐2. The results indicated that knocking down RDM1 led to a significant increase of the percentage of annexin V‐positive fractions. (B) Cell cycle analysis for si*RDM1* SHSY5Y and SK‐N‐AS cells. These data showed significantly increased cell populations in the G0/G1 phase and decreased cell populations in the G2/M phase of RDM1‐silenced NB cells.

Growth signaling pathways to promote cell proliferation are associated with dysregulated apoptosis, and so we next analyzed cell apoptosis in *RDM1*‐silenced SH‐SY5Y and SK‐N‐AS cells. As expected, the results showed that *RDM1* knockdown cells had more apoptotic populations than siN control cells (Fig. 3A).

Inhibition of siRDM1 growth may also be due to cell cycle arrest; therefore, cell cycle analysis was performed by flow cytometry. These data showed significantly increased cell populations in the G0/G1 phase and decreased cell populations in the G2/M phase of RDM1‐silencing SH‐SY5Y cells (Fig. 3B). Similar effects were observed in SK‐N‐AS cells (Fig. 3B). These results suggested that the growth inhibition in siRDM1 cells might be associated with the cell apoptosis and G2/M cell cycle arrest.

### Silencing *RDM1* inhibits *in vivo* growth of NB cells

**Figure 4:**
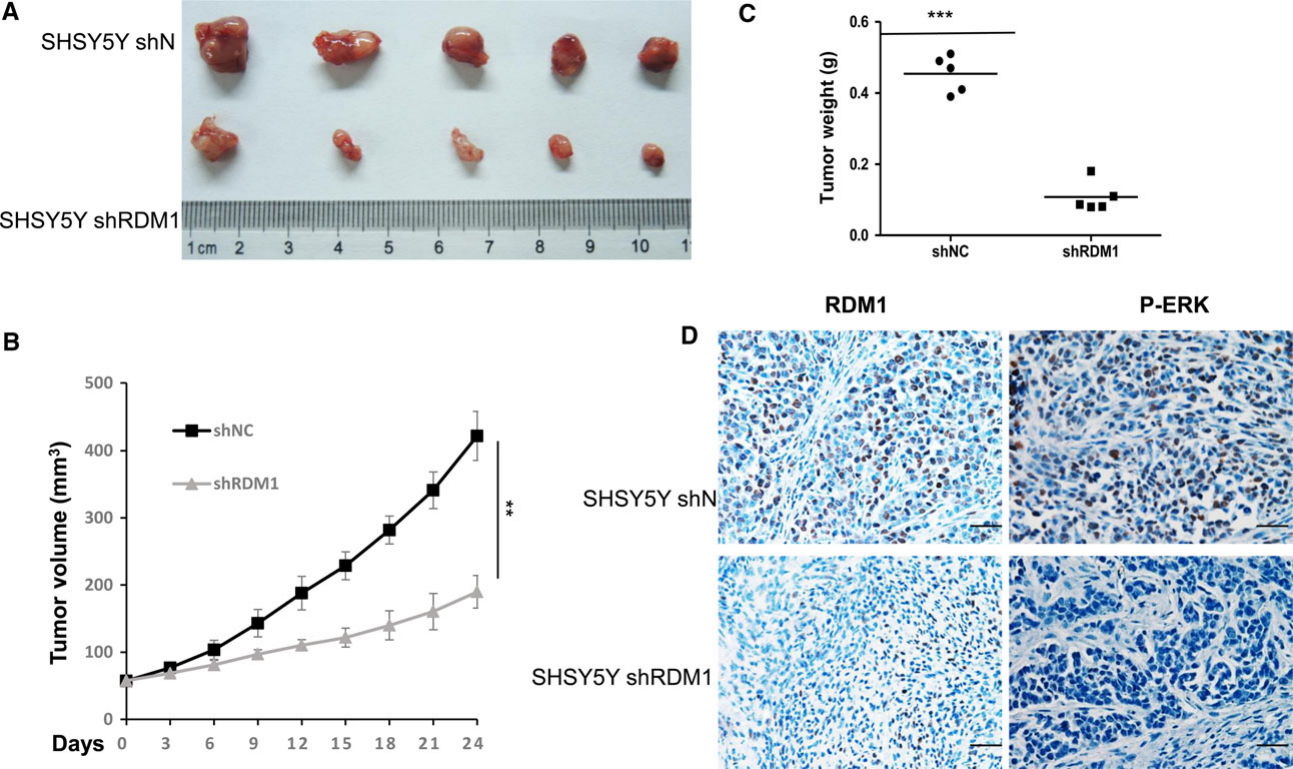
Silencing *RDM1* inhibits *in vivo* growth of NB cells. (A) Representative xenograft tumors originated from stable *RDM1*‐knockdown (sh*RDM1*) SHSY5Y cell. RDM1 depletion resulted in markedly decreased tumor growth. (B) Standard tumor growth curve described in subcutaneous xenografts. **P <* *0.01. Data represent means ± SEM, as determined by Student's *t* test. (C) The weights of tumors. The tumor weight in shRDM1 cells was less than that from the shN cells. Data represent means ± SEM, as determined by Student's *t* test. ***P <* *0.001. (D) IHC staining of RDM1 and P‐ERK in *RDM1*‐knockdown (shRDM1) or control (shN) tumors. The results showed that P‐ERK decreased in *RDM1* knockdown tumors. Scale bar: 25 μm (magnification: ×40).

To further examine whether RDM1 has an oncogenic role *in vivo*, we generated the stable *RDM1*‐knockdown cell line of SH‐SY5Y (SH‐SY5Y sh*RDM1*). We subcutaneously injected SH‐SY5Y shN control and shRDM1 cells into flanks of BALB/C nude mice. As shown in Fig. 4A, RDM1 depletion resulted in markedly decreased tumor growth. Furthermore, the tumor size and weight in sh*RDM1* cells was less than that from the shN cells (Fig. 4B,C), confirming the oncogenic role of RDM1 in NB progression. A low frequency of missense mutations of genes in the Ras–Raf–MEK–ERK pathway has been reported in NB and activation of ERK1/2 signaling has frequently been observed in NB 19, 20, 21. Further confirming the RDM1 mechanism involved, IHC data demonstrated that RDM1 is an important regulator of P‐ERK by down‐regulating P‐ERK at the protein level (Fig. 4D).

### RDM1 promotes cell proliferation by regulating RAS–Raf–MEK–ERK signaling pathway

**Figure 5:**
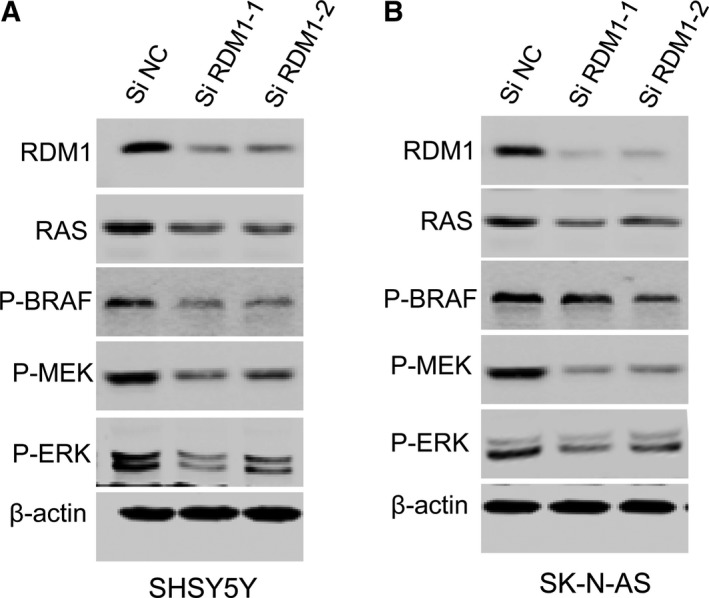
RDM1 regulates RAS–Raf–MEK–ERK signaling pathway in human NB cells. (A,B) Western blots of RDM1, RAS, P‐BRAF, P‐MEK and P‐ERK in si*RDM1* SH‐SY5Y (A) and SK‐N‐AS (B) cells. The results showed that knockdown of *RDM1* significantly decreased the level of RAS, P‐BRAF, P‐MEK and P‐ERK.

To examine how the endogenous RDM1 affects the expression of P‐ERK, we carried out a western blotting assay to determine the protein level of the Ras–Raf–MEK–ERK pathway in siRDM1 and siN cell. Our results showed that knockdown of *RDM1* significantly decreased the level of RAS, P‐BRAF, P‐MEK and P‐ERK in SH‐SY5Y and SK‐N‐AS *siRDM1* cells (Fig. 5A,B). Therefore, knocking down *RDM1* in NB cells suppresses cell growth, strongly confirming the idea that RDM1 regulated the RAS–Raf–MEK–ERK signaling pathway.

## Discussion

In this study, we identified a novel oncogene, RDM1, mediating the progression of NB, supporting the role of RDM1 in cell proliferation and tumorigenesis. Since DNA repair protein RAD52 has previously been implicated in the development of resistance to cancer therapy 22, RDM1 was initially indicated to have a similar function to RAD52 in DNA repair pathways 16. Our Oncomine‐based (TCGA database) expression analyses and IHC staining in clinical tumor samples confirmed that RDM1 was high expressed.

Previous work showed the regulation of RDM1 in lung cancer 16 and papillary thyroid carcinoma 17. However, in this study, we identified RDM1 as an oncogenic target in NB. Firstly, we found knockdown of *RDM1* could reduce NB cell proliferation, augment cell apoptosis and induce cell cycle arrest. Furthermore, the xenograft mouse model showed stable knockdown of *RDM1* significantly inhibits NB tumor growth. These results served as direct evidence that RDM1 might be of benefit for cancer cell survival. Studies of the role of DNA repair in the maintenance of genome integrity in mammalian cells have provided insight into the molecular aspects in cancer 23, and incorrectly repaired DSBs could cause mutations or chromosome rearrangements and result in cancer progression 24. Our finding of the increased cell apoptosis and cell cycle arrest in siRDM1 cells provides a potential mechanistic basis for the impaired DSB response. Future work should define whether DDR pathways will be altered in the context of manipulating RDM1.

Despite multimodal therapy recently, NB patients still have a poor prognosis. Therefore, exploring the mechanisms will provide potentially novel therapeutic targets for NB patients. The RAS–RAF–MEK–ERK pathway is important for regulation of cell proliferation, survival, differentiation and migration in different tumors including NB 25, 26. Previous work showed that the combination of AZD8055 and MEK inhibitor U0126 enhanced the cell growth inhibition of NB cells *in vitro* and *in vivo* 26, indicating the importance of this pathway. Our discovery demonstrated that RDM1 may regulate the expression of MEK/ERK *in vitro* and *in vivo*, as *RDM1*‐knockdown cells showed significant down‐regulation of P‐ERK and P‐MEK. Therefore, we predict that RDM1 can be a potential prognostic marker for NB because of its regulation of MEK/ERK signals. However, whether regulation of RDM1–ERK is the only mechanism remains for further study.

In summary, in this study, we reveal an oncogenic role of RDM1 in NB. Our clinical data also show overexpression of RDM1 in NB samples. Before our study, there were no reports for RDM1 regulation of the RAS–Raf–MEK–ERK signaling pathway during NB cancer progression, and this work also show that RDM1 may be a target for antineoplastic therapies.

## Conflict of interest

The authors declare no conflict of interest.

## Author contributions

GX and HW participated in the design of the study and performed the measurements and the statistical analysis. WC, MC and WH helped in data collection and the interpretation of data. WY and YC wrote the manuscript. All authors read and approved the manuscript.
